# Atelocollagen Increases Collagen Synthesis by Promoting Glycine Transporter 1 in Aged Mouse Skin

**DOI:** 10.3390/ijms262411825

**Published:** 2025-12-07

**Authors:** Kyung-A Byun, Seung Min Oh, Seyeon Oh, Hyoung Moon Kim, Myungjune Oh, Geebum Kim, Min Seung Kim, Kuk Hui Son, Kyunghee Byun

**Affiliations:** 1 Department of Anatomy and Cell Biology, College of Medicine, Gachon University, Incheon 21936, Republic of Korea; 2 LIBON Inc., Incheon 22006, Republic of Korea; 3 GangnamON Clinic, Seoul 06129, Republic of Korea; 4 Functional Cellular Networks Laboratory, Lee Gil Ya Cancer and Diabetes Institute, Gachon University, Incheon 21999, Republic of Korea; 5 Maylin Clinic, Goyang 10391, Republic of Korea; 6 Misogain Dermatology Clinic, Gimpo 10108, Republic of Korea; 7 Inee Clinic, Seoul 06030, Republic of Korea; 8 Department of Thoracic and Cardiovascular Surgery, Gil Medical Center, Gachon University, Incheon 21565, Republic of Korea; 9 Department of Health Sciences and Technology, Gachon Advanced Institute for Health Sciences and Technology, Gachon University, Incheon 21999, Republic of Korea

**Keywords:** atelocollagen, collagen, glycine transporter 1

## Abstract

Aging results in decreased collagen synthesis and the disruption of extracellular matrix integrity, primarily due to increased oxidative stress. This study evaluated whether atelocollagen can restore collagen synthesis in aged skin by modulating glycine transporter 1 (GlyT1)-mediated glycine uptake, regulating oxidative stress, and influencing extracellular matrix remodeling factors in senescent human cells and the skin of older mice. Human dermal fibroblasts (HDFs) and aged mouse skin were treated with atelocollagen, with analyses of GlyT1 expression, glutathione and intracellular glycine concentrations, oxidative stress markers, nuclear factor-kappa-B (NF-κB) activity, matrix metalloproteinases (MMPs), SMAD proteins (SMADs) signaling, and collagen I/III. Treatment with GlyT1 inhibitors and glycine was tested. Atelocollagen significantly increased GlyT1 expression and intracellular glycine concentration, promoted glutathione synthesis, and reduced oxidative stress. These effects led to decreased NF-κB activity and MMP1/3/9 expression, increased SMAD2/3 phosphorylation, and upregulated type I/III collagen synthesis in senescent HDFs and aged mouse skin. All beneficial effects were blocked by GlyT1 inhibition and were generally superior to glycine alone. Histology showed increased collagen density and improved skin elasticity in atelocollagen-treated mice. In summary, atelocollagen enhances collagen synthesis and reduces oxidative stress in aged skin through GlyT1-dependent glycine transport, providing a potential strategy for skin rejuvenation.

## 1. Introduction

Collagen, the most common protein in animals, makes up approximately 75% of the dry weight of skin. It forms a triple helix structure, with a repeating primary amino acid sequence of glycine-proline-X or glycine-X-hydroxyproline. In this pattern, X can be any amino acid except for glycine, proline, or hydroxyproline [1].

Human skin typically contains several types of collagen, with collagen I accounting for 80–90%, collagen III for 8–12%, and collagen V for 5% [2]. Type I collagen is the primary structural component responsible for tensile strength, whereas type III collagen contributes to tissue elasticity and flexibility within the extracellular matrix. Type V collagen plays a regulatory role in fibrillogenesis by controlling fibril diameter and initiating collagen fiber assembly [3].

As a key component of the dermal extracellular matrix (ECM), collagen offers structural support and is vital for keeping skin firm and elastic [4]. A hallmark of both natural aging and photoaging is an alteration in both the quantity and structure of collagen [5].

By natural aging or photoaging, reactive oxygen species (ROS) concentrations increase in the skin. An increase in ROS production and a decrease in the activity of antioxidant enzymes disrupt the balance of oxidative stress [6]. Excessive free radicals activate the nuclear factor-kappa-B (NF-κB) signaling pathway, which is a key contributor to the rise in matrix metalloproteinase (MMP) expression [7]. Matrix metalloproteinases are capable of almost completely degrading the various protein components of the ECM, including collagen, thereby progressively destroying the integrity of the dermis [7]. Additionally, excessive ROS and transcription factor Jun production inhibit the transforming growth factor beta (TGF-β) signaling pathway, which eventually reduces type I collagen synthesis [8,9]. Moreover, upregulation of NF-κB activates Smad7 (mothers against decapentaplegic homolog 7), which eventually inhibits Smad2 and Smad3 [10]. Increased ROS decreases phosphorylation of Smad3, which leads to decreased type I collagen expression [11].

This aging process leads to a decline in the collagen I to collagen III ratio, which can reduce skin tension, elasticity, and wound healing [12]. For these reasons, controlling collagen production is important for both medical and cosmetic applications [13].

The main function of fibroblasts is to synthesize collagen. This ability is directly affected by the availability of the specific amino acids required for collagen’s construction, such as glutamine, glycine, and proline [14,15,16,17]. In dermal fibroblasts, glycine is superior to proline, glutamine, and leucine at increasing collagen synthesis [1]. However, in articular chondrocytes, administration of either proline, lysine, or glycine increases collagen synthesis [17]. Therefore, creating targeted treatments for aging dermis requires knowledge about glycine and its effects.

Glycine also acts as an antioxidant and provides protective effects in various organs [18,19,20]. Its cytoprotective function is linked to increased activity of the glycine receptor (GlyR) and glycine transporter 1 (GlyT1) [21,22]. Glycine transporter 1 has a strong affinity for glycine and moves it into the cytosol [21]. This process is important because glycine is a necessary component for creating glutathione, one of the body’s primary endogenous antioxidants [23]. Glycine supplements activate GlyT1, which facilitates the transport of glycine into the cytosol and boosts glutathione synthesis [24]. An increase in ROS creates a positive feedback loop that activates nicotinamide adenine dinucleotide phosphate (NADPH) oxidase (NOX), a major source of ROS, leading to a vicious cycle that amplifies oxidative stress [25]. Through GlyR activation, glycine inhibits NOX, reducing oxidative stress [24]. Glycine also lowers oxidative stress in pancreatic β-cells, protecting against high-glucose-induced cell damage [24].

Historically, purified collagens from human or animal sources have been used as injectable fillers to immediately restore decreased dermal volume. While this approach provided instant aesthetic improvement, a key drawback was the short-term duration of the effect due to the rapid enzymatic degradation of the injected collagen within the tissue [26]. Another drawback of using animal-derived collagen is its potential to trigger an immune or allergic reaction when injected into humans. To overcome this limitation, atelocollagen was developed. Atelocollagen is created by using type I pepsin to remove the N- and C-terminal telopeptides from collagen. These telopeptides are responsible for causing an antigenic, or immune, response in humans; thus, their removal makes atelocollagen a safer option for injection [27,28].

Atelocollagen injections have been reported to increase collagen synthesis in the skin and cartilage [29,30]. However, the exact mechanism by which atelocollagen increases collagen synthesis is not yet fully understood.

Based on the known antioxidant effects of glycine and evidence that a reduction in ROS can decrease collagen-degrading MMPs while increasing collagen synthesis through the SMAD2/3 pathway, we hypothesize that when atelocollagen is injected into the skin, it is first broken down into glycine, which then reduces intracellular ROS, thereby increasing collagen synthesis and decreasing its degradation.

To evaluate this hypothesis, we investigated whether atelocollagen increases GlyT1 expression in both a senescent human fibroblast model and aged mouse skin. We sought to confirm whether the resultant increase in cytosolic glycine would promote glutathione synthesis, leading to a reduction in ROS and a subsequent decrease in NOX expression. Furthermore, we evaluated whether this reduction in ROS would diminish NF-κB activity, thereby decreasing the expression of MMP1, 3, and 9 to reduce collagen degradation. Concurrently, we aimed to determine whether this process decreases SMAD7 expression, which increases the phosphorylation of SMAD2/3 and ultimately increases collagen synthesis.

## 2. Results

### 2.1. Atelocollagen Increases GlyT1 Expression and Intercellular Glycine Concentration in Senescent Human Fibroblasts

Given our hypothesis that injected atelocollagen is enzymatically broken down into glycine within tissues before acting on cells, we first sought to confirm if the same degradation process occurs in in vitro cell culture. This validation was necessary to justify our planned mouse model experiments. To address the potential for confounding results, as the cell culture medium Dulbecco’s modified Eagle medium (DMEM) already contains glycine, we compared glycine concentrations across four conditions: DMEM alone, DMEM with atelocollagen, medium from cultures of human dermal fibroblasts (HDFs), and medium from HDFs treated with atelocollagen. Although there was no significant difference in glycine concentration among the DMEM (GM), DMEM with atelocollagen (GM wAtCOL), and HDF-only medium conditions (CM), the medium from HDFs treated with atelocollagen (CM wAtCOL) showed an almost three-fold increase in glycine concentration compared with the other conditions. In addition, the atelocollagen solution used in this study (150 μg/mL) contained approximately 3 mM glycine, as demonstrated by our compositional analysis (Figure S1). This finding provides indirect evidence that when atelocollagen is administered to cells, they break it down into glycine.

We established a senescent cell model by treating HDFs with hydrogen peroxide, confirming the induction of senescence by an increase in the expression of senescence markers p16 and p21 (Figure S2).

The concentration of atelocollagen for the cell experiments was chosen to be a concentration that increases GlyT1 expression without compromising cell viability. Cell viability was found to decrease at an atelocollagen concentration of 15 mg/mL (Figure S3A). Although the expression of GlyT1 messenger RNA (mRNA) increased with rising atelocollagen concentrations, there was no significant difference between 150 μg/mL and 300 μg/mL. This saturation of effect suggests that concentrations greater than 150 μg/mL offer no additional benefit. Therefore, a concentration of 150 μg/mL of atelocollagen was selected for all subsequent cell experiments (Figure S3B).

To investigate whether atelocollagen treatment of senescent HDFs increases GlyT1 expression and consequently raises intracellular glycine concentration, we planned an experiment to confirm a decrease in intracellular glycine concentration after treating the cells with ALX5407, a selective GlyT1 inhibitor [31]. To find an appropriate concentration of ALX5407 for our experiments, we treated senescent HDFs with varying concentrations of ALX5407 and observed a dose-dependent decrease in intracellular glycine concentration. As there was no further change in intracellular glycine concentration at ALX5407 concentrations of 200 nM and higher, we used 200 nM of ALX5407 for subsequent experiments (Figure S3C).

**Figure 1:**
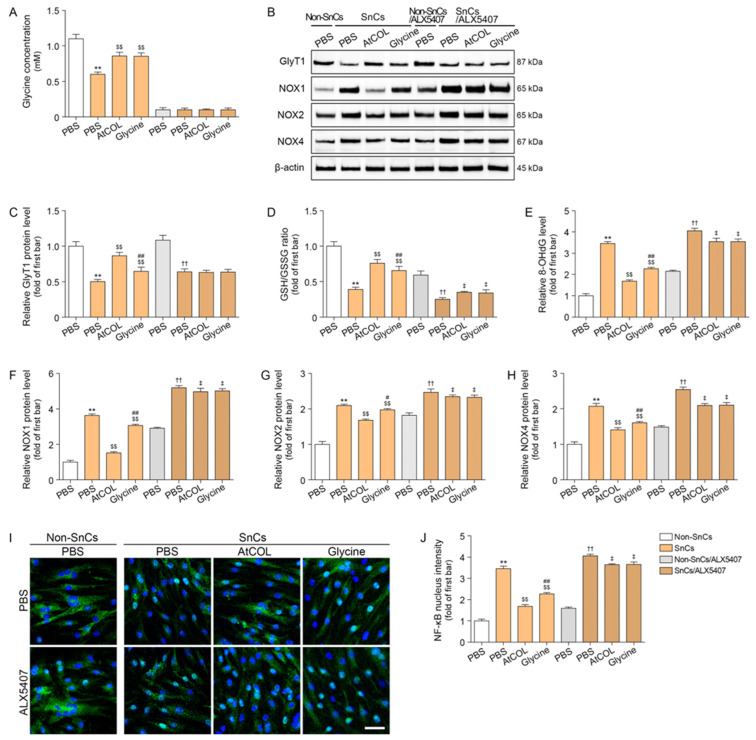
Atelocollagen regulates glycine, GlyT1, and oxidative stress in ALX5407-dependent senescent human dermal fibroblasts. (**A**) Glycine concentration significantly increased after atelocollagen treatment in senescent cells; this effect was suppressed by ALX5407 in senescent fibroblasts. (**B**,**C**) Western blot analysis showed that the expression levels of GlyT1 increased after atelocollagen treatment. β-actin served as the loading control. (**D**) Relative GSH/GSSG ratio significantly increased after atelocollagen treatment. (**E**) Enzyme-linked immunosorbent assay results show reduced 8-OHdG after atelocollagen treatment. (**B**,**F**–**H**) Western blot analysis showed that the expression levels of NOX1/2/4 decreased after atelocollagen treatment. β-actin served as the loading control. (**I**,**J**) Representative immunocytochemistry images for NF-κB localization in senescent fibroblasts (scale bar = 50 μm). Nuclei are stained with DAPI (blue), and the NF-κB signal is shown in green. Data are expressed as the mean ± standard deviation from biological replicates (N = 5 per group). **, *p* < 0.01 (SnCs/PBS vs. Non-SnCs/PBS); $$, *p* < 0.01 (SnCs/AtCOL or Glycine vs. SnCs/PBS); #, *p* < 0.05 and ##, *p* < 0.01 (SnCs/Glycine vs. SnCs/AtCOL); ††, *p* < 0.01 (SnCs/ALX5407/PBS vs. Non-SnCs/ALX5407/PBS); ‡, *p* < 0.05 (SnCs/ALX5407/AtCOL or Glycine vs. SnCs/ALX5407/PBS) (Mann–Whitney U test). 8-OHdG, 8-hydroxy-2-deoxyguanosine; ALX5407, selective GlyT1 inhibitor; AtCOL, atelocollagen; GlyT1, glycine transporter 1; GSH, reduced glutathione; GSSG, oxidized glutathione; NF-κB, nuclear factor-kappa-B; Non-SnCs, non-senescent cells; Non-SnCs/ALX5407, non-senescent cells pretreated with ALX5407; NOX, nicotinamide adenine dinucleotide phosphate oxidase; PBS, phosphate-buffered saline; SnCs, senescent cells; SnCs/ALX5407, senescent cells pretreated with ALX5407.

We hypothesized that the glycine contained in atelocollagen is the primary effective factor for collagen synthesis. Therefore, we compared the effects of atelocollagen to those of a single treatment with glycine in HDFs. As the atelocollagen at 150 μg/mL contains 3 mM of glycine, we performed the experiment using 3 mM glycine. Intracellular glycine concentration decreased in senescent HDFs, and it increased after atelocollagen and glycine treatment. The increase was inhibited by ALX5407 (Figure 1A). Upon cellular senescence induced by hydrogen peroxide treatment, GlyT1 expression decreased. Subsequent treatment with either atelocollagen or glycine ameliorated the reduced GlyT1 expression. However, the extent to which atelocollagen restored GlyT1 expression was greater than that of glycine. Furthermore, this restorative effect was eliminated when GlyT1 was inhibited by ALX5407 (Figure 1B,C).

### 2.2. Atelocollagen Decreases Oxidative Stress and NF-κB Activity in Senescent Human Fibroblasts

Increased glycine can be used for glutathione synthesis, which in turn reduces intracellular oxidative stress. To confirm this, we assessed the increase in glutathione synthesis by measuring the ratio of reduced glutathione (GSH) and oxidized glutathione (GSSG) and determined the level of intracellular oxidative stress by measuring 8-hydroxy-2-deoxyguanosine (8-OHdG) concentration. Furthermore, to investigate whether increased oxidative stress led to a rise in NOX signaling, we evaluated the expression levels of NOX1, NOX2, and NOX4 via Western blotting.

Decreased GSH/GSSG was observed in senescent HDFs. Treatment with atelocollagen or glycine both increased this ratio, but the effect was more pronounced with atelocollagen. However, the increase in GSH/GSSG induced by both atelocollagen and glycine was diminished when the cells were treated with ALX5407 (Figure 1D).

Increased 8-OHdG concentration was observed in senescent HDFs. Treatment with atelocollagen or glycine both reduced this concentration, but the effect was more pronounced with atelocollagen. However, the reduction in 8-OHdG concentration induced by both atelocollagen and glycine was diminished when the cells were treated with ALX5407 (Figure 1E).

Expression of NOX1/2/4 increased in senescent HDFs and was decreased by atelocollagen or glycine treatment. These decreasing effects were more prominent after treatment with atelocollagen than with glycine. However, the decreased effect diminished when the cells were treated with ALX5407 (Figure 1B,F–H).

In this study, NF-κB activity was evaluated via intranuclear intensity of NF-κB. NF-κB activity increased in senescent HDFs, and it was decreased by atelocollagen or glycine treatment. The decrease was more prominent in cells treated with atelocollagen than with glycine. However, those decreasing effects diminished when the cells were treated with ALX5407 (Figure 1I,J).

### 2.3. Atelocollagen Decreases MMP1/3/9 Expression and Increases SMAD2/3 Expression in Senescent Human Fibroblasts

**Figure 2:**
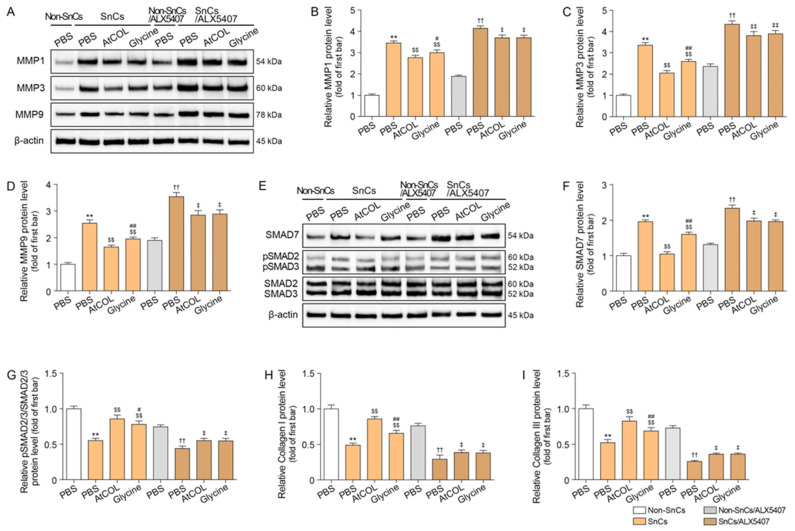
Atelocollagen regulates collagen degradation and synthesis in ALX5407-dependent senescent fibroblasts. (**A**–**D**) Western blot analysis showed that the expression levels of MMPs decreased after atelocollagen treatment. β-actin served as the loading control. (**E**–**G**) Western blot analysis showed that the expression levels of SMAD7 and phosphorylated SMAD2/3 (pSMAD2 Ser465/467, pSMAD3 Ser423/425) regulated after atelocollagen treatment. β-actin served as the loading control. (**H**,**I**) Enzyme-linked immunosorbent assay results show increased collagen I and collagen III after atelocollagen treatment. Data are expressed as the mean ± standard deviation from biological replicates (N = 5 per group). **, *p* < 0.01 (SnCs/PBS vs. Non-SnCs/PBS); $$, *p* < 0.01 (SnCs/AtCOL or Glycine vs. SnCs/PBS); #, *p* < 0.05 and ##, *p* < 0.01 (SnCs/Glycine vs. SnCs/AtCOL); ††, *p* < 0.01 (SnCs/ALX5407/PBS vs. Non-SnCs/ALX5407/PBS); ‡, *p* < 0.05 and ‡‡, *p* < 0.01 (SnCs/ALX5407/AtCOL or Glycine vs. SnCs/ALX5407/PBS) (Mann–Whitney U test). ALX5407, selective GlyT1 inhibitor; AtCOL, atelocollagen; MMP, matrix metalloproteinase; Non-SnCs, non-senescent cells; Non-SnCs/ALX5407, non-senescent cells pretreated with ALX5407; p, phosphorylated; PBS, phosphate-buffered saline; SMAD, mothers against decapentaplegic homolog; SnCs, senescent cells; SnCs/ALX5407, senescent cells pretreated with ALX5407.

Increased the total levels of MMP1/3/9 was observed in senescent HDFs. Treatment with atelocollagen or glycine both reduced MMP1/3/9 expression, but the effect was more pronounced with atelocollagen. However, the reduction in MMP1/3/9 expression induced by both atelocollagen and glycine was diminished when the cells were treated with ALX5407 (Figure 2A–D).

SMAD7 expression increased in senescent HDFs, and it was decreased by atelocollagen or glycine treatment. The decreased effect was more prominent in the cells treated with atelocollagen than with glycine. However, this decrease diminished when the cells were treated with ALX5407 (Figure 2E,F). Decreased expression of phosphorylated SMAD2/3 was observed in senescent HDFs. Treatment with atelocollagen or glycine both increased this expression, but the effect was more pronounced with atelocollagen. However, the increase in phosphorylated SMAD2/3 induced by both atelocollagen and glycine was diminished when the cells were treated with ALX5407 (Figure 2E,G).

Decreased expression of collagen I and III was observed in senescent HDFs. Treatment with atelocollagen or glycine both increased this expression, but the effect was more pronounced with atelocollagen. However, the increase in collagen I and III induced by both atelocollagen and glycine was diminished when the cells were treated with ALX5407 (Figure 2H,I).

### 2.4. Atelocollagen Increases GlyT1 Expression and Decreases Oxidative Stress in Aged Mouse Skin

**Figure 3:**
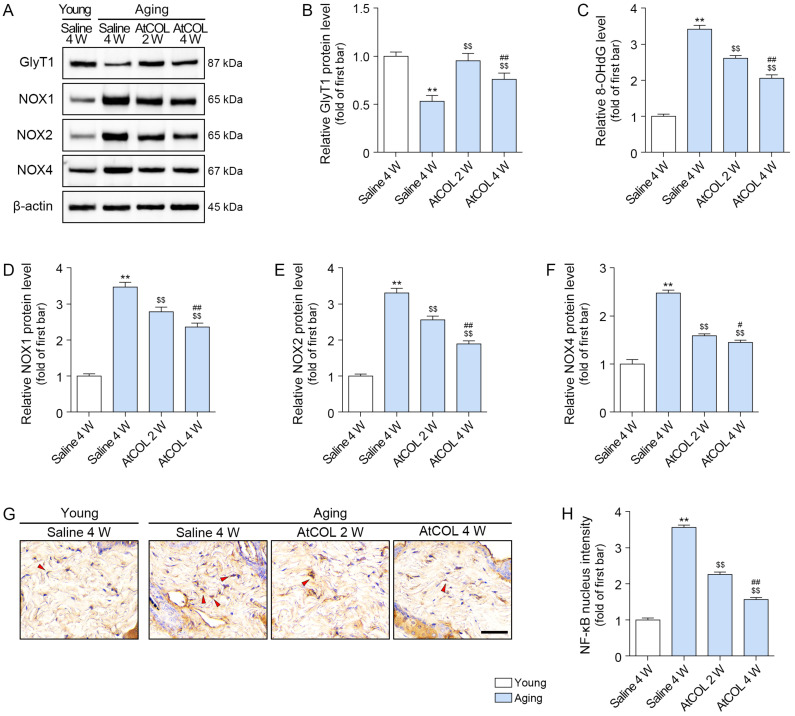
Atelocollagen regulates GlyT1 expression and oxidative stress in aged mouse skin. (**A**,**B**) Western blot analysis showed that the expression levels of GlyT1 increased after atelocollagen treatment. β-actin served as the loading control. (**C**) Enzyme-linked immunosorbent assay results show reduced 8-OHdG concentration after atelocollagen treatment. (**A**,**D**–**F**) Western blot analysis showed that the expression levels of NOX1/2/4 decreased after atelocollagen treatment. β-actin served as the loading control. (**G**,**H**) Representative immunohistochemistry images for NF-κB nucleus intensity in aged mouse skin (scale bar = 50 μm). The red marks were positive signals. Data are expressed as the mean ± standard deviation from biological replicates (N = 5 per group). **, *p* < 0.01 (Aging/Saline vs. Young/Saline); $$, *p* < 0.01 (Aging/AtCOL vs. Aging/Saline); #, *p* < 0.05 and ##, *p* < 0.01 (Aging/AtCOL 4W vs. Aging/AtCOL 2W) (Mann–Whitney U test). 8-OHdG, 8-hydroxy-2-deoxyguanosine; AtCOL, atelocollagen; GlyT1, glycine transporter 1; NF-κB, nuclear factor-kappa-B; NOX, nicotinamide adenine dinucleotide phosphate oxidase; W, week.

In aged mouse skin, atelocollagen increased GlyT1 expression, with the increasing effect greatest at 2 weeks after injection (Figure 3A,B).

The concentration of 8-OHdG and the expression of NOX1/2/4 increased in aged skin, and atelocollagen decreased these effects (Figure 3A,C–F). NF-κB activity increased in aged skin and decreased after atelocollagen treatment. This decrease was greatest at 4 weeks after injection (Figure 3G,H).

### 2.5. Atelocollagen Decreases MMP1/3/9 Expression and Increases SMAD2/3 and Collagen Expression in Aged Mouse Skin

**Figure 4:**
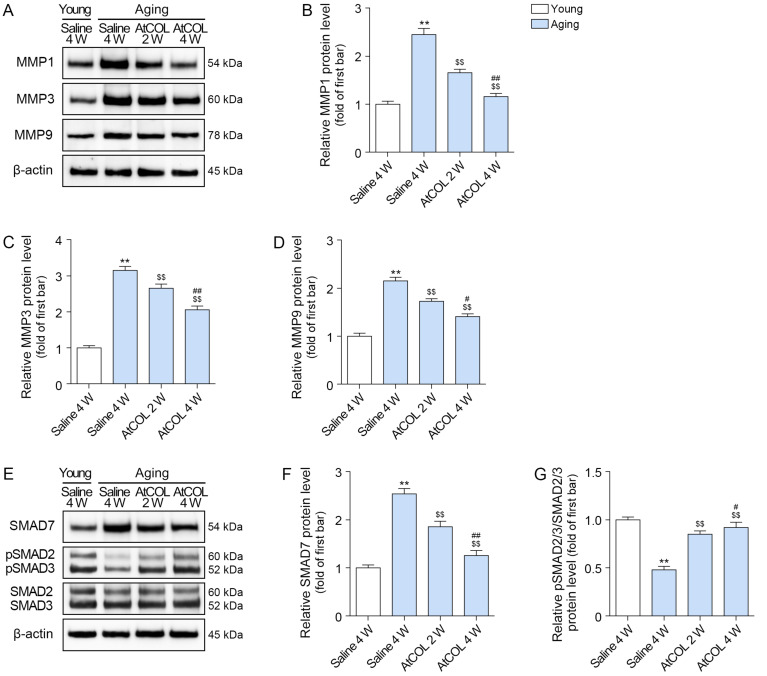
Atelocollagen regulates collagen degradation and synthesis in aged mouse skin. (**A**–**D**) Western blot analysis showed that the expression levels of MMPs decreased after atelocollagen treatment. β-actin served as the loading control. (**E**–**G**) Western blot analysis showed that the expression levels of SMAD7 and phosphorylated SMAD2/3 (pSMAD2 Ser465/467, pSMAD3 Ser423/425) regulated after atelocollagen treatment. β-actin served as the loading control. Data are expressed as the mean ± standard deviation from biological replicates (N = 5 per group). **, *p* < 0.01 (Aging/Saline vs. Young/Saline); $$, *p* < 0.01 (Aging/AtCOL vs. Aging/Saline); #, *p* < 0.05 and ##, *p* < 0.01 (Aging/AtCOL 4W vs. Aging/AtCOL 2W) (Mann–Whitney U test). AtCOL, atelocollagen; MMP, matrix metalloproteinase; p, phosphorylated; SMAD, mothers against decapentaplegic homolog; W, week.

Expression of total MMP1/3/9 and SMAD7 increased in aged skin and decreased after atelocollagen treatment. This decrease was greatest at 4 weeks after injection. Expression of phosphorylated SMAD2/3 decreased in aged skin and increased after atelocollagen treatment. This increase was greatest at 4 weeks after injection (Figure 4).

**Figure 5:**
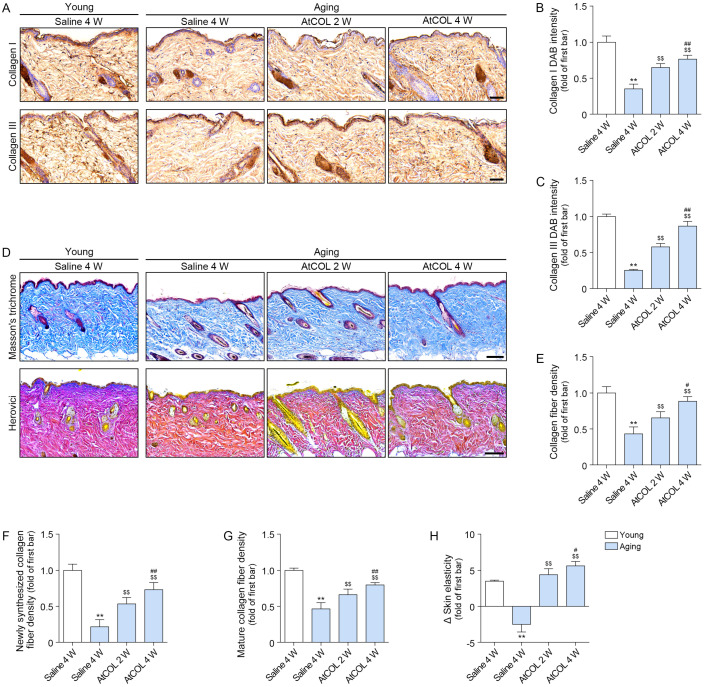
Atelocollagen enhances collagen synthesis, fiber density, and skin elasticity in aged mouse skin. (**A**) Representative immunohistochemistry images of collagen I and collagen III expression after atelocollagen treatment (scale bar = 50 μm). (**B**,**C**) Quantification of DAB intensity for collagen I (**B**) and collagen III (**C**), respectively. (**D**,**E**) Masson’s trichrome staining for visualization of collagen fibers (blue) in the dermis (scale bar = 100 μm). (**D**–**G**) Herovici staining is used to distinguish newly synthesized collagen fibers (blue) and mature collagen fibers (red) in the dermis (scale bar = 100 μm). (**H**) Skin elasticity after atelocollagen treatment. Data are expressed as the mean ± standard deviation from biological replicates (N = 5 per group). **, *p* < 0.01 (Aging/Saline vs. Young/Saline); $$, *p* < 0.01 (Aging/AtCOL vs. Aging/Saline); #, *p* < 0.05 and ##, *p* < 0.01 (Aging/AtCOL 4W vs. Aging/AtCOL 2W) (Mann–Whitney U test). AtCOL, atelocollagen; DAB, 3,3′-diaminobenzidine; W, week.

The expression of collagen I and III decreased in aged skin and increased after atelocollagen treatment. This increase was greatest at 4 weeks after injection (Figure 5A–C).

Collagen density, evaluated by Masson’s trichrome staining, decreased in aged skin and increased after atelocollagen treatment. This increase was greatest at 4 weeks after injection (Figure 5D,E).

Herovici staining was conducted to differentiate between mature collagen, which stains red, and newly formed collagen, which stains blue [32,33]. Both newly synthesized and mature collagen were decreased in aged skin and increased after atelocollagen treatment. This increase was greatest at 4 weeks after injection (Figure 5D,F,G).

Skin elasticity was evaluated using an API 100 Skin Analyzer (Aram Huvis, Seongnam, Republic of Korea). Skin elasticity was decreased in aged skin and increased after atelocollagen treatment. The increase was greatest at 4 weeks after injection (Figure 5H).

## 3. Discussion

**Figure 6:**
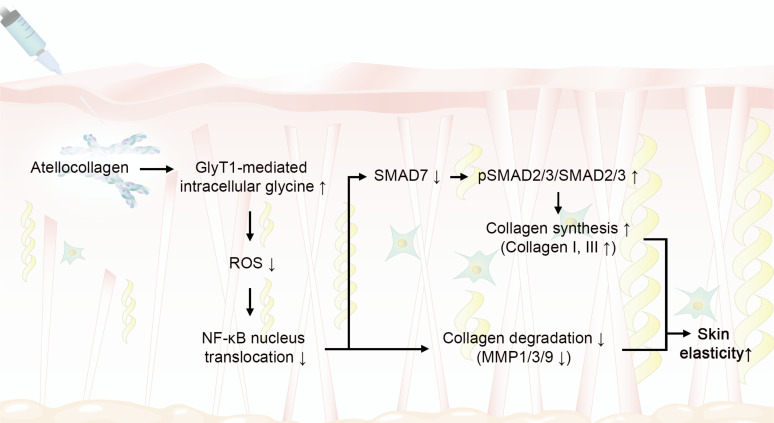
Graphical diagram by which atelocollagen restores collagen homeostasis with glycine transporter 1 in aged skin. The up (↑) and down (↓) arrows indicate increased or decreased expression, respectively.

This study aimed to determine whether atelocollagen can restore collagen synthesis in aged skin by increasing intracellular glycine through GlyT1-mediated uptake and subsequently reducing oxidative stress. We found that atelocollagen markedly upregulated GlyT1 expression in senescent human fibroblasts and aged mouse skin. These changes were accompanied by reduced oxidative stress, decreased NOX and NF-κB activity, and lower expression of total MMP1/3/9. In parallel, atelocollagen increased SMAD2/3 phosphorylation and restored type I and III collagen production, leading to improved collagen fiber density and skin elasticity in aged mice (Figure 6).

Age-related physiological changes markedly alter dermal collagen composition, leading to a reduction in ECM density and fragmentation of type I collagen, accompanied by a relative increase in type III collagen [34,35]. These structural changes reduce skin elasticity and firmness, resulting in wrinkles and facial sagging [34,35]. Therefore, improving age-related skin changes requires not only volumetric augmentation but also stimulation of collagen synthesis and restoration of an organized collagen network within the ECM.

Collagen-derived proteins such as gelatin and collagen peptides contain abundant proline and glycine and have been shown to stimulate collagen synthesis in various tissues [36,37,38]. According to multiple reports, oral collagen supplements improve skin elasticity in humans [39]. Furthermore, collagen peptides increase collagen fiber content and improve skin laxity in aged animals [40].

Dermal collagen fillers can provide immediate volumetric correction but have limited duration compared with synthetic materials [41]. If the breakdown products of injected collagen can themselves promote collagen synthesis, then the filler could serve a dual function: providing immediate volume augmentation while simultaneously enhancing the ECM structure by stimulating collagen production.

For this reason, we sought to determine whether glycine, one of the primary breakdown products of injected collagen, could increase collagen synthesis. Glycine, a fundamental component of collagen, reduces oxidative stress, a key factor in accelerating aging. Therefore, we hypothesized that if glycine can mitigate oxidative stress, particularly in aged skin, it could further enhance the benefits of collagen fillers.

Collagen is degraded to glycine by enzymes involved in intracellular and extracellular degradation pathways [42]. In this study, we observed that treating HDFs with atelocollagen increased glycine concentration within the cell culture medium. Although we did not precisely determine the specific enzymatic mechanism responsible for atelocollagen degradation, the elevated glycine concentration in the atelocollagen-treated HDF culture medium—compared with both HDFs cultured alone and atelocollagen incubated in medium without cells—strongly indicates that HDFs actively degrade atelocollagen, leading to the release of free glycine.

Glycine serves as a key precursor for glutathione synthesis [43,44]. By facilitating glycine transport into the cytosol, GlyT1 is essential for the synthesis of glutathione, a potent antioxidant [21]. Consequently, the ability of glycine to reduce oxidative stress is dependent on the proper functioning of GlyT1 [21]. Exogenous glycine has been applied to human intestinal Caco-2 and HCT-8 cells prior to an oxidative challenge with tert-butyl hydroperoxide, which effectively protected the cells by decreasing intracellular ROS [21]. However, the protective effect was blocked when a specific GLYT1 inhibitor was used [21]. Previous studies have demonstrated that glycine reduces oxidative stress primarily by increasing glutathione synthesis, a process dependent on the enhanced function of GlyT1 for transporting glycine into the cytosol [21]. Building on this finding, we hypothesized that the glycine generated from the degradation of atelocollagen would similarly increase intracellular glycine concentration by enhancing GlyT1 function.

Our experimental results support this hypothesis, showing that atelocollagen treatment increased intracellular glycine levels in senescent HDFs. The co-treatment with a GlyT1 inhibitor decreased intracellular glycine, confirming that the elevated glycine concentration is a direct result of increased glycine transport mediated by GlyT1. Furthermore, we observed that atelocollagen increased not only GlyT1 function but also its protein expression, which was initially reduced in the senescent cells.

This finding contrasts with previous research, which reported that glycine treatment does not significantly alter GlyT1 mRNA expression in cells [44]. However, another study showed that glycine increased expression of GlyT1 in PEC-1 cells during 4-HNE–induced oxidative stress. The precise mechanism by which atelocollagen increases GlyT1 protein expression in aged cells, and the reason for this discrepancy with previous findings that showed GlyT1 expression did not increase by glycine treatment, warrants further investigation.

Our results show that atelocollagen treatment increased the GSH/GSSG ratio and decreased 8-OHdG. These antioxidative effects were attenuated by GlyT1 inhibition. Atelocollagen also reduced NOX1/2/4 and NF-κB activity and decreased MMPs, while increasing Smad2/3 and collagen types I and III; all effects were diminished by GlyT1 inhibition. Interestingly, although GlyT1 inhibition reduced intracellular glycine across all groups, differences in oxidative stress and NOX expression persisted. This suggests an additional glycine-mediated mechanism, potentially via glycine receptors (GlyRs), which reduce NOX function when stimulated by extracellular glycine [24]. Because GlyRs were not examined here, future studies should clarify their involvement. Furthermore, although Western blot analysis identified changes in MMPs protein levels, the antibodies used in this study are not isoform-specific; therefore, the results reflect total MMP expression rather than a distinction between pro- and active forms. Isoform-specific or activity-based analyses may further refine these findings in future studies. Additionally, the ability of atelocollagen to reduce oxidative stress and decrease MMP expression was more pronounced than the ability of glycine alone. This finding suggests that atelocollagen is likely to contain other peptides, in addition to glycine, that contribute to its antioxidative properties, such as proline [45].

The primary objective of this study was to test the hypothesis that atelocollagen exerts its effects through its glycine content, so we did not investigate which other specific components within atelocollagen may reduce oxidative stress. This aspect warrants further investigation.

The mouse study results showed similar trends: increased GlyT1 expression, decreased NOX and NF-κB activity, MMPs, and increased Smad2/3 and collagen types I/III, with increased collagen density persisting up to 4 weeks.

This study did not aim to determine the precise residence time of atelocollagen in the skin or the long-term duration of its collagen-generating effects. We observed that although GlyT1 expression peaked at 2 weeks post-injection, the reduction in oxidative stress and the increase in collagen synthesis were more pronounced at 4 weeks than at 2 weeks post-injection. This finding suggests that the positive effects of glycine, such as reducing oxidative stress and expression of NF-κB and MMPs, may be sustainable over a longer period. The long-term duration of these effects, however, should be confirmed in future studies.

This study has several limitations that should be acknowledged. First, although we observed that atelocollagen increases GlyT1 protein expression in senescent human fibroblasts and aged mouse skin, the upstream regulatory mechanisms responsible for this change remain unclear. We did not examine whether atelocollagen or its degradation products directly influence known transcriptional regulators of GlyT1, such as NF-κB, SP1, or Nrf2, nor did we perform promoter–reporter assays that could clarify potential transcriptional activation. As a result, the mechanism underlying GlyT1 upregulation remains speculative and requires further investigation. Second, while GlyT1 inhibition attenuated the antioxidant and collagen-promoting effects of atelocollagen, we did not conduct complementary gain-of-function studies, such as GlyT1 overexpression, nor did we examine loss-of-function models using genetic knockdown. These experiments would help define the causal role of GlyT1 in mediating the biological effects observed in this study and will be important for validating the proposed pathway. Third, we did not characterize the enzymatic processes responsible for the degradation of atelocollagen into free glycine, nor did we assess whether different forms of collagen (e.g., telocollagen) might exhibit distinct degradation kinetics or cellular responses. These factors may influence the magnitude or duration of glycine-mediated effects and merit further examination. Finally, our study did not assess long-term safety, immune responses, or the persistence of atelocollagen in vivo beyond the 4-week observation period. Additional research, including extended animal studies and clinical investigations, will be necessary to determine the duration, safety, and translational relevance of the findings.

Despite these limitations, our research demonstrates that atelocollagen can increase intracellular glycine concentration, leading to a reduction in oxidative stress and an amelioration of the diminished collagen synthesis capacity of senescent cells. This study’s novelty lies in its finding that atelocollagen increases intracellular glycine levels via GlyT1, which eventually increases collagen synthesis.

## 4. Materials and Methods

### 4.1. Atelocollagen Preparation

Atelocollagen was supplied by Dmed, LLC (Gyeonggi, Republic of Korea). The atelocollagen was extracted and purified from the skin of a 6-month-old specific-pathogen-free miniature pig as described earlier [46,47]. The process involved cleaning, decontaminating, degreasing, and slicing the pig skin into thin pieces. The minced skin was acidified using hydrochloric acid and treated with pepsin to selectively hydrolyze the non-helical telopeptides and other non-collagenous proteins, preserving the integrity of the triple-helical collagen structure. The resulting hydrolyzed gel was dissolved in phosphate-buffered saline (PBS), and the pH was adjusted to a range of 6 to 8 using 10 M sodium hydroxide. The gel was then filtered sequentially through 300 kDa and 100 kDa cut-off filters (MilliporeSigma, Burlington, MA, USA).

The purified type I atelocollagen gel (3% *w*/*v*, 150 kDa) was sterilized using a low-temperature steam sterilization method in a steam sterilization chamber (HS-1000R, Hanshinmed, Seoul, Republic of Korea). The final collagen gel for this study was pre-filled into 3 mL syringes (Laetigen, 2 mL per syringe).

Each production batch passed rigorous quality control checks, including tests for pH, collagen content, enzymatic resistance, and sterility, conducted at the Dmed GMP facility. The quality control procedures were performed as follows:

(1) pH: pH was measured using a calibrated pH meter. The electrode was rinsed with distilled water and calibrated with pH 4.0 and pH 7.0 standard buffers, then immersed directly into the test sample. The acceptable pH range was 6.5–7.5.
(2) Collagen content: Collagen content was quantified by hydroxyproline analysis. One gram of sample was hydrolyzed in 35% HCl at 110 °C, diluted to 1 mg/mL, and analyzed using a hydroxyproline assay kit. Collagen concentration was calculated using the hydroxyproline-based formula described in the internal standard, with an acceptance range of 90.0–110.0% of the indicated amount (3% *w*/*v*).
(3) Enzymatic resistance: Sample pieces (10 mm × 10 mm) were immersed in trypsin solution and shaken at 37 °C for 5 h. Structural integrity was evaluated by comparing residual mass and morphology with reference collagen. Samples were required not to degrade within 24 h.
(4) Sterility: Sterility was assessed according to ISO 17665 [48]. Samples were incubated in appropriate culture media and monitored for microbial growth. Batches were accepted only when no microbial contamination was observed.

### 4.2. In Vitro Experiments

#### 4.2.1. Cell Culture and Senescence Induction

Human dermal fibroblasts (CEFO^TM^ Human Dermal Fibroblast Cells; CEFO Co., Ltd., Seoul, Republic of Korea) were used between passages 5 and 8. Cells were cultured in CEFOgro^TM^ Human MSC Growth Medium (CEFO Co., Ltd.) and maintained at 37 °C in a humidified incubator with 5% CO_2_. For senescence induction, HDFs were seeded at 1 × 10^6^ cells in 100-mm dishes and cultured for 48 h, followed by treatment with hydrogen peroxide (350 μM; Sigma-Aldrich, St. Louis, MO, USA) for 1.5 h. After treatment, cells were washed with Dulbecco’s PBS (DPBS; Thermo Fisher Scientific, Waltham, MA, USA) to remove residual hydrogen peroxide, and the medium was replaced with fresh growth medium. Cells were further incubated for 72 h before sample collection [49]. In parallel, a control group was treated with DPBS under the same conditions. Samples were harvested for RNA isolation to confirm the induction of cellular senescence.

#### 4.2.2. Cytotoxicity Assessment of Atelocollagen

To evaluate atelocollagen cytotoxicity, senescent HDFs were seeded at 5 × 10^4^ cells per well in 96-well plates and allowed to attach overnight. Cells were then treated with atelocollagen at concentrations of 0.1, 0.5, 1, 5, 10, and 15 mg/mL for 48 h. Atelocollagen working concentrations were prepared by diluting the stock solution in DPBS and subsequently mixing the diluted atelocollagen with serum-free medium to achieve the final treatment concentrations. Cell viability was measured using the TransDetect Cell Counting Kit (TransGen Biotech Co., Ltd., Beijing, China), and the non-toxic concentration range was determined.

#### 4.2.3. Determination of Working Concentration of Atelocollagen

Based on cytotoxicity results, hydrogen peroxide–induced senescent HDFs were treated with atelocollagen at concentrations of 0, 50, 150, and 300 μg/mL for 48 h to determine the optimal non-toxic dose. Similarly, working concentrations were prepared by diluting the stock solution in DPBS and subsequently mixing the diluted atelocollagen with serum-free medium to achieve the final treatment concentrations. From these conditions, 150 μg/mL atelocollagen was selected as the working concentration for subsequent experiments. Control groups included DPBS-treated non-senescent cells and hydrogen peroxide–treated senescent cells without atelocollagen.

#### 4.2.4. Preparation of Conditioned Medium

To evaluate glycine release from atelocollagen under cell culture conditions, four types of media were prepared: (1) growth medium without cells (GM), (2) GM supplemented with atelocollagen without cells, (3) conditioned medium collected after culturing HDFs for 48 h (CM), and (4) conditioned medium collected after culturing HDFs with atelocollagen for 48 h. The collected media were concentrated using Amicon Ultra centrifugal filter units (MilliporeSigma) at 3500 rpm for 45 min prior to analysis.

#### 4.2.5. Inhibition Study in Senescent Human Dermal Fibroblasts Treated with Atelocollagen and Glycine

To assess the inhibitory effects of atelocollagen and glycine, with or without ALX5407 [31], senescent HDFs were divided into eight experimental groups:

(1) Non-senescent cells treated with DPBS (Non-SnCs/PBS)
(2) Senescent cells treated with DPBS (SnCs/PBS)
(3) Senescent cells treated with atelocollagen (150 μg/mL) (SnCs/AtCOL)
(4) Senescent cells treated with glycine (3 mM) (SnCs/Glycine)
(5) Non-senescent cells pretreated with ALX5407 (200 nM) and treated with DPBS (Non-SnCs/ALX5407/PBS)
(6) Senescent cells pretreated with ALX5407 (200 nM) and treated with DPBS (SnCs/ALX5407/PBS)
(7) Senescent cells pretreated with ALX5407 (200 nM) and treated with atelocollagen (150 μg/mL) (SnCs/ALX5407/AtCOL)
(8) Senescent cells pretreated with ALX5407 (200 nM) and treated with glycine (3 mM) (SnCs/ALX5407/Glycine)

ALX5407 (200 nM; Sigma-Aldrich), a selective inhibitor of GlyT1, was prepared by diluting the stock solution in DPBS and administered 24 h prior to atelocollagen or glycine treatment. Cells were then exposed to atelocollagen (150 μg/mL) or glycine (3 mM) for 48 h. Atelocollagen and glycine were likewise prepared by diluting the respective stock solutions in DPBS and subsequently mixing them with serum-free medium to achieve the final treatment concentrations, with control groups receiving equivalent volumes of DPBS.

### 4.3. In Vivo Experiments

#### 4.3.1. Experimental Design

We used transparent porcine type 1 atelocollagen provided by PHARVIS KOREA (Seoul, Republic of Korea). Six-week-old C57BL/6N mice were purchased from Orient Bio Inc. (Seongnam, Republic of Korea). After mating the mice, the offspring were allowed to age naturally. At 15 months of age, male mice were randomly divided into four groups for experimental treatment (N = 5 per group) [50]. This study was conducted by the Gachon University Animal Experiment Ethics Committee (Institutional Animal Care and Use Committee approval number LCDI-2025-0001).

Porcine type 1 atelocollagen was injected into aged mice, and changes were observed for 2 or 4 weeks. As a comparison group, young mice (4 months old) and aged mice (15 months old) were injected with the same amount of saline solution.

Group 1: Young mice were injected with saline, and tissues were collected at 4 weeks (Young/Saline group).

Group 2: Aged mice were injected with saline, and tissues were collected at 4 weeks.

Group 3: Aged mice were injected with atelocollagen, and tissues were collected at 2 weeks.

Group 4: Aged mice were injected with atelocollagen, and tissues were collected at 4 weeks.

Mice were anesthetized and had their hair removed using a razor and depilatory cream, and then the material was injected. The syringe was used for injection as provided. The amount of material injected was similar to that used in typical human clinical trials, with a per-surface area ratio of 0.7 mL/4 cm^2^.

#### 4.3.2. Skin Elasticity Measurement

Skin elasticity was assessed using an API 100 Skin Analyzer (Aram Huvis). High-resolution images of the skin surface were captured using a non-contact optical method, and elasticity was quantified using the accompanying software. All mice were measured five times immediately prior to sampling, and the average value was calculated.

### 4.4. RNA Extraction and Quantitative Real-Time PCR

RNA was extracted using RNAiso Plus reagent (Takara Bio Inc., Shiga, Japan), following the manufacturer’s instructions. Complementary DNA (cDNA) was synthesized from 1 μg of total RNA using the PrimeScript 1st strand cDNA synthesis kit (Takara Bio Inc.). Quantitative real-time PCR was performed using SYBR Green PCR Master Mix (Takara Bio Inc.), gene-specific primers and synthesized cDNA. Amplification and detection were carried out with the QuantStudio Real-Time PCR system (Thermo Fisher Scientific). The relative mRNA expression levels were normalized to β-actin (*Actb*), and data were analyzed using the comparative ΔΔCt method.

### 4.5. Protein Isolation and Western Blot Analysis

Samples were lysed using a RIPA lysis kit (ATTO Corporation, Tokyo, Japan) supplemented with phosphatase and protease inhibitors. Protein concentrations were determined using the Pierce BCA protein assay kit (Thermo Fisher Scientific).

Equal amounts of protein (30 μg per sample) were separated by sodium dodecyl sulfate–polyacrylamide gel electrophoresis and transferred onto polyvinylidene difluoride membrane (MilliporeSigma). Membranes were blocked with 5% skim milk in Tris-buffered saline containing 0.1% Tween 20 for 1 h at room temperature and incubated overnight at 4 °C with the appropriate primary antibodies (Table S1). After washing, membranes were incubated with horseradish peroxidase (HRP)-conjugated secondary antibodies (Vector Laboratories, Newark, CA, USA) for 1 h at room temperature. Protein bands were visualized using Amersham ECL detection reagents (Cytiva, Marlborough, MA, USA) and quantified by densitometric analysis with ImageJ software (version 1.53s; National Institutes of Health, Bethesda, MD, USA). Target protein expression was normalized to β-actin, and the normalized values were expressed as fold changes relative to the Non-SnCs/PBS or Young/Saline groups.

### 4.6. Glycine Quantification and Glutathione/Oxidized Glutathione Ratio Analysis

Glycine concentrations in cell and tissue lysates and concentrated conditioned medium were measured using a glycine enzyme-linked immunosorbent assay kit (Biorbyt, Cambridge, UK) in accordance with the manufacturer’s instructions.

Reduced glutathione and GSSG concentrations in cell and tissue lysates were quantified using a GSH/GSSG assay kit (Abcam, Cambridge, UK) in accordance with the manufacturer’s instructions.

### 4.7. Immunocytochemistry

Cells were prepared as described for cell culture and senescence induction. After treatment under the indicated conditions, cells were fixed with 4% paraformaldehyde in PBS for 15 min at room temperature and washed three times with PBS. For permeabilization, cells were incubated with 0.5% Triton X-100 in PBS for 10 min, followed by blocking with normal serum blocking solution (Vector Laboratories) for 1 h at room temperature. Cells were incubated overnight at 4 °C with the appropriate primary antibodies (Table S1) diluted in blocking solution. After washing, Alexa Fluor–conjugated secondary antibodies (Invitrogen, Thermo Fisher Scientific) were applied for 1 h at room temperature in the dark. Nuclei were counterstained with 4′,6-diamidino-2-phenylindole (DAPI; Sigma-Aldrich) for 10 s, and coverslips were mounted with DPX mounting medium (Sigma-Aldrich). Fluorescent images were acquired using an LSM 710 confocal laser scanning microscope (Carl Zeiss Microscopy GmbH, Jena, Germany) at the Core Facility for Cell to In Vivo Imaging (Gachon university, Incheon, Republic of Korea), and signal intensities were analyzed using ZEN software (version 3.1; Carl Zeiss Microscopy GmbH, Jena, Germany).

### 4.8. Preparation of Paraffin-Embedded Skin Tissue Blocks

The skin tissues were fixed in 4% paraformaldehyde at 4 °C for 72 h. After fixation, tissues were transferred into tissue cassettes and washed with distilled water in situ. The fixed tissues were then dehydrated through a graded ethanol series (Duksan Pure Chemicals Co., Ltd., Ansan, Republic of Korea), cleared in xylene (Duksan Pure Chemicals Co., Ltd.), and embedded in paraffin (Leica, Wetzlar, Germany). Paraffin blocks were sectioned into 7-µm thick slices using a microtome (Thermo Fisher Scientific), mounted on coated slides (Muto Pure Chemicals Co., Ltd., Tokyo, Japan), and incubated overnight at 60 °C for subsequent histological analyses.

#### 4.8.1. Immunohistochemistry

For immunohistochemical staining, paraffin-embedded sections were deparaffinized in xylene and rehydrated through a graded ethanol series. Permeabilization was performed using 0.5% Triton X-100 for 5 min, followed by additional PBS washes. To block non-specific binding, sections were incubated with normal serum blocking solution for 1 h at room temperature. Slides were then incubated overnight at 4 °C with the primary antibody (Table S1) diluted in blocking solution. After washing, sections were incubated with biotin-conjugated secondary antibody (Vector Laboratories) for 1 h at room temperature. Immunoreactivity was visualized using the ABC reagent (Vector Laboratories) following the manufacturer’s protocol, and nuclei were counterstained with hematoxylin (KPNT, Cheongju, Republic of Korea). Finally, sections were dehydrated, cleared in xylene, and mounted with DPX mounting medium. Stained tissues were scanned using a Motic Scan Infinity 100 slide scanner (Motic, Beijing, China), and representative images were captured for analysis. Quantification of staining positive for 3,3′-diaminobenzidine (DAB) was performed using ImageJ software. Specifically, the density of nuclei stained brown within the dermis was measured, and normalized values were expressed as fold changes relative to the Young/Saline group.

#### 4.8.2. Masson’s Trichrome Staining

Masson’s trichrome staining was performed using a commercial Trichrome Stain Kit (Modified Masson’s) (ScyTek Laboratories, Logan, UT, USA) in accordance with the manufacturer’s instructions. Stained slides were dehydrated, mounted, and scanned using a slide scanner, and collagen fiber density was quantified using ImageJ software. Specifically, collagen-positive areas (blue) within the dermis were isolated using color deconvolution, and the density of collagen-stained area was measured and normalized values were expressed as fold changes relative to the Young/Saline group.

#### 4.8.3. Herovici Staining

Herovici staining was carried out using a commercial Herovici Stain Kit (ScyTek Laboratories) in accordance with the manufacturer’s instructions. Sections were stained with Weigert’s iron hematoxylin followed by Herovici solution, then dehydrated and mounted. Stained slides were scanned using a slide scanner, and representative images were captured. The density of newly synthesized collagen fibers (blue) and mature collagen fibers (red) were quantified separately using ImageJ software [32,33]. Specifically, young collagen (blue) and mature collagen (red) were separated using color deconvolution, and the density of each collagen subtype was measured and normalized values were expressed as fold changes relative to the Young/Saline group.

### 4.9. Quantitative and Statistical Analysis

Quantitative data are presented as mean ± standard deviation. Although data distribution was assessed using the Shapiro–Wilk test, the sample size of each group is insufficient to reliably evaluate normality or homogeneity of variance. Therefore, non-parametric statistical methods were used throughout all analyses. The Kruskal–Wallis test was used followed by pairwise comparisons using the Mann–Whitney U test. All statistical analyses were performed using SPSS version 26 (IBM, Armonk, NY, USA). Statistical significance and biological replicates are indicated in the legends of each figure.

## 5. Conclusions

In this study, we demonstrate that atelocollagen enhances collagen synthesis in aged skin models through a mechanism involving increased intracellular glycine availability and restoration of GlyT1 function. These changes contribute to improved redox balance, reduced oxidative-stress–related signaling, the suppression of collagen-degrading enzymes, and reactivation of SMAD2/3. These changes ultimately result in normalization of collagen I/III expression and improvement in dermal collagen accumulation. While atelocollagen is clinically used for soft-tissue augmentation, the present findings pertain specifically to its cellular and biochemical effects, and further studies will be required to determine whether these mechanistic benefits translate into clinical outcomes or structural tissue changes in vivo.

## Data Availability

The original contributions presented in this study are included in the article/Supplementary Materials. Further inquiries can be directed to the corresponding author(s).

## Supplementary Materials

The supporting information can be downloaded at https://www.mdpi.com/article/10.3390/ijms262411825/s1.
