# Supplementary material for: Atelocollagen Increases Collagen Synthesis by Promoting Glycine Transporter 1 in Aged Mouse Skin
Source: Int J Mol Sci. 2025 Dec 7;26(24):11825. doi: 10.3390/ijms262411825 (PMC12732917; doi:10.3390/ijms262411825)
Supplement: Supplementary file 1 [file ijms-26-11825-s001.zip › ijms-3970561-supplementary.pdf]

Table S1. List of antibodies for enzyme-linked immunosorbent assay (ELISA), western blot (WB) and immunocytochemistry (ICC) / immunohistochemistry (IHC).

Antibody	Company	Dilution rate		
		ELISA	WB	ICC/IHC
β-actin	Cell signaling	-	1:1,000	-
GlyT1	Bioryt	-	1:1,000	-
8-OHdG	Abcam	1:500	-	-
NOX1	Affinity	-	1:1,000	-
NOX2	Affinity	-	1:1,000	-
NOX4	Affinity	-	1:1,000	-
NF-κB	Cell signaling	-	-	1:200
MMP1	FINETEST	-	1:500	-
MMP3	ABclonal	-	1:500	-
MMP9	ABclonal	-	1:500	-
SMAD7	Santa cruz	-	1:500	-
SMAD2/3	Cell signaling	-	1:1,000	-
pSMAD2/3	Cell signaling	-	1:1,000	-
Collagen I	Santa cruz	1:500	-	1:50
Collagen III	Santa cruz	1:500	-	1:50

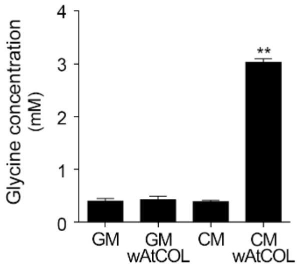

Figure S1. Confirmation of glycine concentration of atelocollagen in DMEM or conditioned media. Data are expressed as the mean ± standard deviation. **, p < 0.01, vs. 1st bar (Mann–Whitney U test). AtCOL, atelocollagen; CM, medium from cultures of HDF; CM wAtCOL, me-dium from HDFs treated with atelocollagen; DMEM, Dulbecco's modified Eagle medium; GM, DMEM alone; GM wAtCOL, DMEM with atelocollagen; HDF, human dermal fibroblasts.

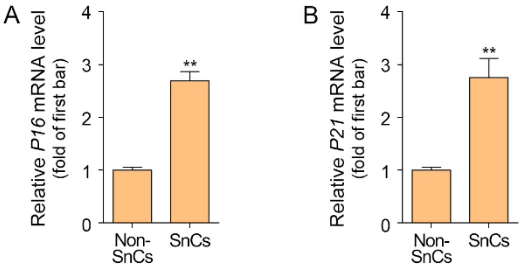

Figure S2. Confirmation of senescence of in H_2O_2-dependent human dermal fibroblasts. (A,B) Changes in *P16* and *P21* RNA expression. Data are expressed as the mean ± standard deviation. **, p < 0.01, vs. 1st bar.

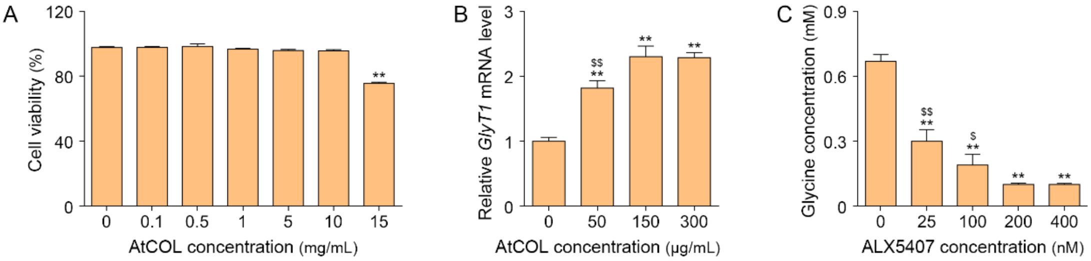

Figure S3. Confirmation of atelocollagen or ALX5407 of optimal concentration in senescent human dermal fibroblasts. (A) Confirmation of cytotoxicity at various concentrations of atelocollagen. (F) Changes in GlyT1 RNA expression following atelocollagen treatment. (G) Changes in glycine concentration following ALX5407 treatment. Data are expressed as the mean ± standard deviation. **, p < 0.01, vs. 1st bar; \$, p < 0.05 and \$\$, p < 0.01, vs. 4th bar (Mann–Whitney U test). ALX5407, selective GlyT1 inhibitor; AtCOL, atelocollagen; GlyT1, glycine transporter 1;.
